# Long-term relief of primary dysmenorrhea following anterior quadratus lumborum block: a case series

**DOI:** 10.3389/fmed.2026.1790019

**Published:** 2026-03-20

**Authors:** Hong Zhu, Yifan Qin, Yi Gu, Jin Wu

**Affiliations:** Department of Anesthesiology, Affiliated Hospital of Jiangsu University, Zhenjiang, Jiangsu, China

**Keywords:** anterior quadratus lumborum block, case report, chronic pelvic pain, primary dysmenorrhea, regional anesthesia

## Abstract

**Background:**

Truncal fascial plane blocks have demonstrated potential efficacy in the treatment of chronic and refractory non-surgical pain. However, reports on the use of fascial plane blocks for the treatment of primary dysmenorrhea are scare.

**Case presentation:**

We reported in three cases that bilateral anterior quadratus lumborum block (AQLB) with 20 ml of 0.375% ropivacaine per side not only provided significant relief from menstrual pain in the month of treatment but also sustained a notable analgesic effect over the subsequent 6 months, manifested as an obvious decrease in Cox Menstrual Symptom Scale score.

**Conclusions:**

AQLB may be an optional alternative for long-term treatment of primary dysmenorrhea.

## Introduction

Primary dysmenorrhea is defined as painful menstruation in the absence of identifiable pelvic pathology. As one of the most prevalent causes of recurring menstrual pain among young women, it can substantially impair quality of life and interfere with daily functioning. Despite its high prevalence, therapeutic options that offer consistent analgesic efficacy without significant adverse effects or potential interference with future reproductive goals of young women remain limited (1). Truncal fascial plane blocks, primarily used for postoperative analgesia, have demonstrated potential efficacy in the treatment of chronic and refractory non-surgical pain (2). Among these techniques, the quadratus lumborum block (QLB) involves the injection of local anesthetic into the fascial planes adjacent to the quadratus lumborum muscle. Its analgesic effect is thought to result from craniocaudal and lateral spread of the injectate, leading to blockade of thoracolumbar nerves traversing these planes (3). Notably, QLB has demonstrated particular efficacy in alleviating visceral abdominal pain. As previously reported, we described a case in which an anterior QLB (AQLB) effectively alleviated drug-resistant primary dysmenorrhea (4). Building on this observation, we subsequently applied bilateral AQLB using the same blocking technique and medications in a series of three additional patients with primary dysmenorrhea (Table 1). All patients had visited the gynecological clinic prior to treatment and were clearly diagnosed with primary dysmenorrhea. To better assess the clinical progression and durability of the analgesic effect, these patients were followed for an observational period of 6 months.

**Table 1:** Demographic and baseline characteristics of the three patients.

**Characteristic**	**Patient 1**	**Patient 2**	**Patient 3**
Age (years)	21	25	19
Height (cm)	165	165	167
Weight (kg)	60	50	58
BMI (kg/m^2^)	22	18.4	20.8
Baseline NRS (0–10)	6–7	7–8	8
Baseline CMSS severity	24	22	20
Baseline CMSS frequency	31	40	28
Treatment timing	Day 1 of menstruation	Day 1 of menstruation	1 day pre-menstruation

NRS, numerical rating scale; CMSS, cox menstrual symptom scale.

## Case report

**Figure 1:**
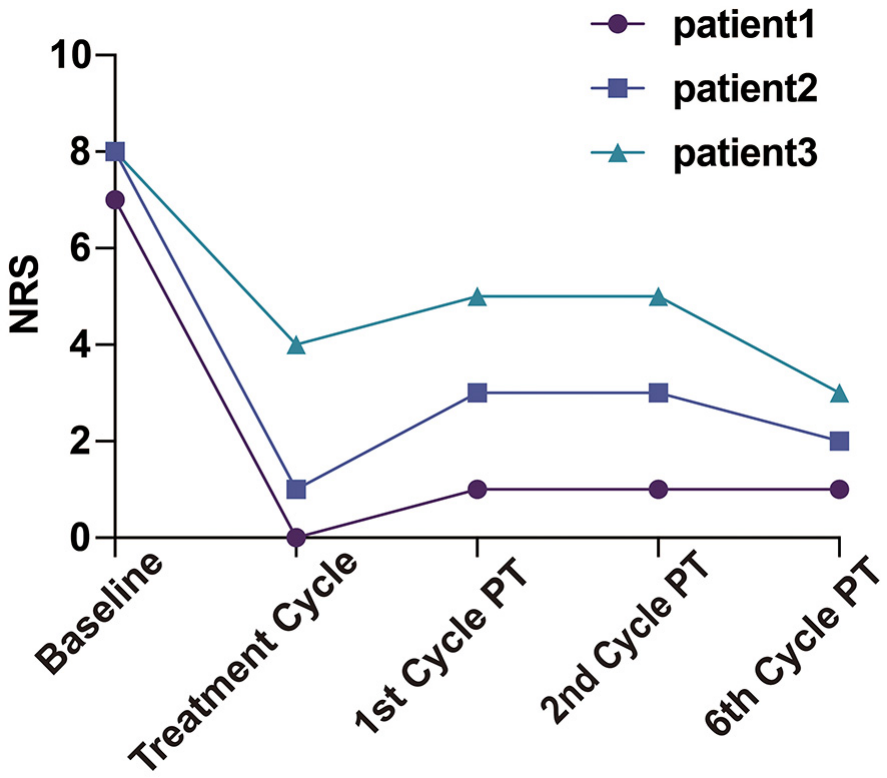
Trends in numerical rating scale (NRS) pain scores for the three patients. PT, post-treatment.

**Figure 2:**
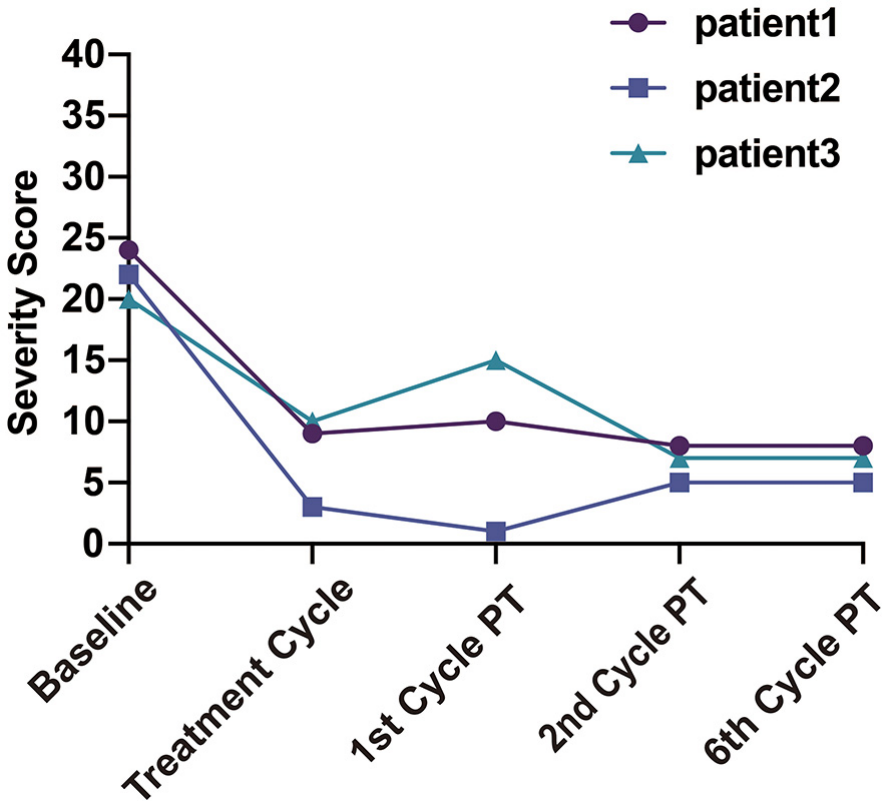
Trends in cox menstrual symptom scale (CMSS) severity scores for the three patients. PT, post-treatment.

**Figure 3:**
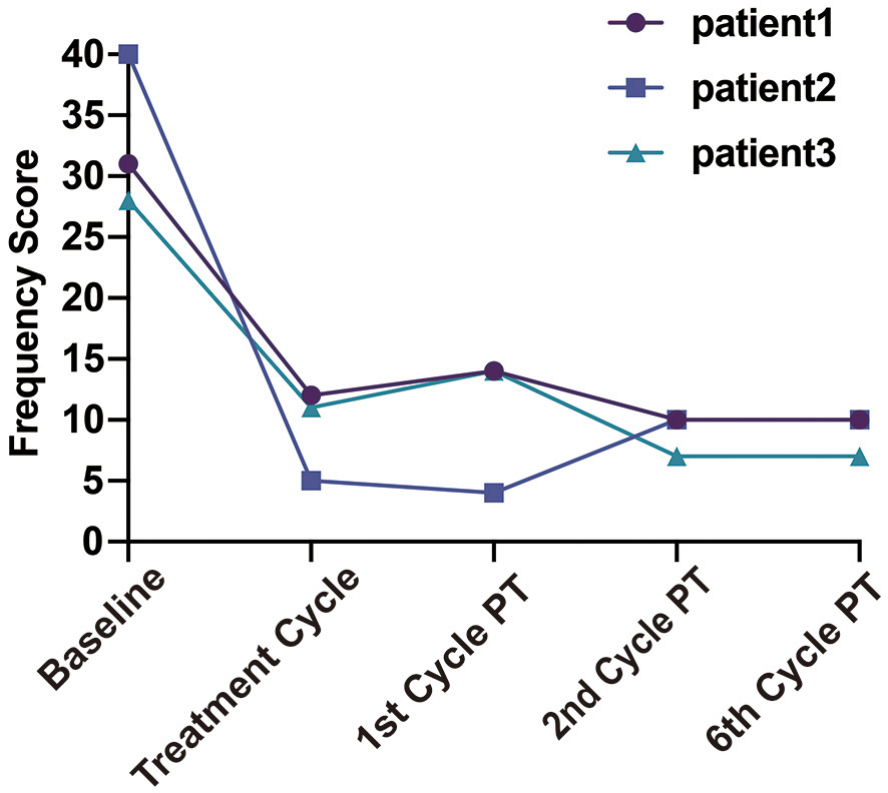
Trends in cox menstrual symptom scale (CMSS) frequency scores for the three patients. PT, post-treatment.

All patients underwent ultrasound-guided bilateral AQLB. The procedure was performed as follows (4, 5): patients were placed in the lateral decubitus position with the target side upward, and the puncture site was marked at the L4 vertebral level. Following routine sterilization and draping, a convex array ultrasound probe (5–8 MHz) covered with a sterile sheath was positioned between the costal margin and the iliac crest to visualize the characteristic “shamrock” sign. Using an in-plane technique, a 22-gauge needle was advanced from posterior to anterior, penetrating the quadratus lumborum muscle (QLM), until the tip reached the plane between the anterior layer of the thoracolumbar fascia and the QLM. Correct needle placement was confirmed by hydrodissection with normal saline, which displaced the QLM away from the needle tip while the psoas major muscle remained stationary. Subsequently, 20 ml of 0.375% ropivacaine was injected, and the identical procedure was repeated on the another side. All blocks were performed by experienced anesthesiologists in a treatment room equipped with emergency resuscitation equipment. Intraoperative monitoring included continuous electrocardiography, non-invasive blood pressure, and pulse oximetry. The total ropivacaine dose of 150 mg (approximately 2.7 mg/kg) remained below the maximum recommended safe limit. Patients were observed for at least 60 min post-block, during which the degree of motor and sensory blockade was assessed and symptoms of local anesthetic systemic toxicity were monitored. Follow-up telephone interviews were conducted at 24 and 72 h post-procedure to evaluate for any signs of adverse reactions. The first two patients were treated on the first day of menstruation, whereas the third patient received treatment 1 day prior to the anticipated onset of menstruation. A blinded researcher collected data by obtaining peak pain Numerical Rating Scale (NRS) and Cox Menstrual Symptom Scale (CMSS) scores via electronic questionnaires at baseline, during the treatment cycle, and at the first, second, and sixth menstrual cycles post-treatment. The demographic characteristics and baseline parameters of the three patients are presented in Table 1. Trends in NRS pain scores and CMSS for the three patients are presented as line graphs in Figures 1–3. No adverse reactions were observed in any of the three patients. This study was conducted in accordance with the Declaration of Helsinki. Written informed consent was obtained from all patients for the publication of this case series.

The first patient was a 21-year-old female (height, 165 cm; weight, 60 kg) who reported abdominal pain during the first 2–3 days of each menstrual cycle, accompanied by lower back pain. The maximum pain intensity reached a NRS score of 6–7. She refrained from taking analgesics due to concerns about potential adverse effects. Following bilateral AQLB, her NRS score decreased to 0 during the same menstrual cycle and remained at a maximum of 1 during subsequent menstruation over the 6-month follow-up period. Compared with pre-treatment levels, the severity score on the CMSS decreased from 24 to 8, and the frequency score decreased from 31 to 10 at 6 months post-treatment (Table 2).

**Table 2:** Cox menstrual symptom scale scores of patient 1.

**Number**	**Symptoms**	**Severity score**					**Frequency score**				
		**Baseline (pre-treatment)**	**Treatment cycle**	**1st cycle post-treatment**	**2nd cycle post-treatment**	**6th cycle post-treatment**	**Baseline (pre-treatment)**	**Treatment cycle**	**1st cycle post-treatment**	**2nd cycle post-treatment**	**6th cycle post-treatment**
1	Abdominal pain	3	0	0	0	0	3	0	0	0	0
2	Nausea	2	1	1	1	1	2	2	1	1	1
3	Vomiting	2	2	1	1	1	2	1	1	1	1
4	Loss of appetite	2	1	1	1	1	3	2	2	2	2
5	Headache	1	0	0	0	0	1	0	0	0	0
6	Back pain	3	0	1	0	0	1	0	1	0	0
7	Leg pain	1	0	0	0	0	3	0	0	0	0
8	Weakness	2	1	1	1	1	4	1	3	1	1
9	Dizziness	2	1	1	1	1	2	2	1	1	1
10	Diarrhea	2	1	1	1	1	3	1	1	1	1
11	Facial blemishes	1	1	1	0	0	2	2	1	0	0
12	Cramps	0	0	0	0	0	0	0	0	0	0
13	Flushing	0	0	0	0	0	0	0	0	0	0
14	Insomnia	0	1	0	0	0	0	1	0	0	0
15	General aching	1	0	0	0	0	2	0	0	0	0
16	Depression	1	0	1	1	1	2	0	2	2	2
17	Irritability	1	0	1	1	1	1	0	1	1	1
18	Nervousness	0	0	0	0	0	0	0	0	0	0
Scores		24	9	10	8	8	31	12	14	10	10

Severity scores: 0-not noticeable, 1-slightly bothersome, 2-moderately bothersome, 3-severely bothersome, 4-very severely bothersome.

Frequency scores: 0-did not occur, 1-lasted less than 3 h, 2-lasted 3–7 h, 3-lasted an entire day, 4-lasted several days.

The second patient was a 25-year-old female (height, 165 cm; weight, 50 kg) who experienced abdominal pain at the onset of menstruation, with an NRS score of 7–8 that gradually subsided over the subsequent days. Prior to treatment, she required four 300-mg tablets of ibuprofen as well as intravenous tramadol (100 mg) during each menstrual period to achieve adequate pain control. Following bilateral AQLB, her NRS score decreased to 1 during the treatment month and remained at 2–3 over the subsequent 6 months. No ibuprofen was used in the treatment month, and analgesic use was reduced to a single 300-mg tablet per menstrual cycle during the 6-month follow-up period. Compared with pre-treatment levels, the severity score of CMSS decreased from 22 to 5, and the frequency score decreased from 40 to 10 at 6 months post-treatment (Table 3).

**Table 3:** Cox menstrual symptom scale scores of patient 2.

**Number**	**Symptoms**	**Severity rating**					**Frequency rating**				
		**Baseline (pre-treatment)**	**Treatment cycle**	**1st cycle post-treatment**	**2nd cycle post-treatment**	**6th cycle post-treatment**	**Baseline (pre-treatment)**	**Treatment cycle**	**1st cycle post-treatment**	**2nd cycle post-treatment**	**6th cycle post-treatment**
1	Abdominal pain	4	0	0	1	1	3	0	0	1	1
2	Nausea	2	1	0	0	0	3	1	0	0	0
3	Vomiting	2	1	0	0	0	3	1	0	0	0
4	Loss of appetite	1	0	0	1	1	3	0	0	3	3
5	Headaches	1	0	0	0	0	1	0	0	0	0
6	Backaches	2	0	0	1	1	4	0	0	1	1
7	Leg aches	0	0	0	0	0	0	0	0	0	0
8	Weakness	2	0	1	1	1	4	0	4	3	3
9	Dizziness	0	1	0	0	0	0	3	0	0	0
10	Diarrhea	3	0	0	0	0	3	0	0	0	0
11	Facial blemishes	1	0	0	0	0	4	0	0	0	0
12	Cramps	0	0	0	0	0	0	0	0	0	0
13	Flushing	0	0	0	0	0	0	0	0	0	0
14	Sleeplessness	0	0	0	0	0	0	0	0	0	0
15	General aching	0	0	0	0	0	0	0	0	0	0
16	Depression	1	0	0	0	0	4	0	0	0	0
17	Irritability	2	0	0	1	1	4	0	0	2	2
18	Nervousness	1	0	0	0	0	4	0	0	0	0
Score		22	3	1	5	5	40	5	4	10	10

Severity ratings: 0-not noticeable, 1-slightly bothersome, 2-moderate bothersome, 3-severely bothersome, 4-very severely bothersome.

Frequency ratings: 0-did not occur, 1-lasted less than 3 h, 2-lasted 3–7 h, 3-lasted an entire day, 4-lasted several days.

The third patient was a 19-year-old female (height, 167 cm; weight, 58 kg) who reported severe abdominal pain beginning 1 day prior to menstruation, which was sufficiently intense to confine her to bed. The peak pain intensity was 8 on NRS. Before treatment, she took two 300-mg tablets of ibuprofen in combination with Yue Shu granules, a traditional Chinese medicine, for symptomatic relief. Following bilateral AQLB, her NRS score decreased to 4 during the treatment month, increased slightly to 5 during the first and second months, and subsequently declined to 3 by 6 months post-treatment. During this period, analgesic requirements were reduced to a single 300-mg tablet of ibuprofen per menstrual cycle. Compared with pre-treatment levels, the severity score of CMSS decreased from 20 to 7, and the frequency score decreased from 28 to 7 at 6 months post-treatment (Table 4).

**Table 4:** Cox menstrual symptom scale scores of patient 3.

**Number**	**Symptoms**	**Severity rating**					**Frequency rating**				
		**Baseline (pre-treatment)**	**Treatment cycle**	**1st cycle post-treatment**	**2nd cycle post-treatment**	**6th cycle post-treatment**	**Baseline (pre-treatment)**	**Treatment cycle**	**1st cycle post-treatment**	**2nd cycle post-treatment**	**6th cycle post-treatment**
1	Abdominal pain	3	2	3	1	1	3	1	1	1	1
2	Nausea	2	1	3	1	1	1	1	1	1	1
3	Vomiting	1	0	1	0	0	1	0	1	0	0
4	Loss of appetite	1	0	1	1	1	1	0	2	1	1
5	Headaches	1	1	1	1	1	1	1	1	1	1
6	Backaches	2	0	1	0	0	4	0	1	0	0
7	Leg aches	2	1	2	1	1	4	1	3	1	1
8	Weakness	1	2	1	1	1	1	3	2	1	1
9	Dizziness	2	1	2	0	0	2	1	1	0	0
10	Diarrhea	1	0	0	0	0	2	0	0	0	0
11	Facial blemishes	1	1	1	0	0	2	2	1	0	0
12	Cramps	0	0	0	0	0	0	0	0	0	0
13	Flushing	0	0	0	0	0	0	0	0	0	0
14	Sleeplessness	0	1	0	0	0	0	1	0	0	0
15	General aching	1	0	0	0	0	3	0	0	0	0
16	Depression	1	0	0	0	0	2	0	0	0	0
17	Irritability	1	0	0	1	1	1	0	0	1	1
18	Nervousness	0	0	0	0	0	0	0	0	0	0
Score		20	10	16	7	7	28	11	14	7	7

Severity ratings: 0-not noticeable, 1-slightly bothersome, 2-moderate bothersome, 3-severely bothersome, 4-very severely bothersome.

Frequency ratings: 0-did not occur, 1-lasted less than 3 h, 2-lasted 3–7 h, 3-lasted an entire day, 4-lasted several days.

## Discussion

Collectively, these cases suggest that AQLB may represent a potential alternative option for the long-term management of primary dysmenorrhea. The sustained analgesic effect observed in these cases may be attributed to the multi-level mechanisms of AQLB. First, from a neuroanatomical perspective, AQLB facilitates the spread of local anesthetic to the lumbar nerve roots and the thoracic sympathetic trunk within the paravertebral space. Such spread may attenuate the transmission of visceral nociceptive signals originating from the uterus (3), thereby reducing uterine ischemia, vasospasm, and the release of inflammatory mediators such as prostaglandins. Superior hypogastric plexus block can relieve chronic pelvic pain and improve associated symptoms such as dysmenorrhea with similar mechanism (6). However, it is more difficult to implement than AQLB. Second, AQLB may exert therapeutic effects on the musculoskeletal component of primary dysmenorrhea. Low back pain, the second most common symptom after lower abdominal pain, may be alleviated through anesthetic infiltration of the thoracolumbar fascia (7). Given that the thoracolumbar fascia is densely innervated with nociceptive fibers, it has been implicated in the pathophysiology of low back pain. Accordingly, AQLB may effectively attenuate local nociceptor sensitization and alleviate mechanical tension (8), thereby contributing to the relief of low back pain. Furthermore, on a systemic level, the persistence of analgesia across several menstrual cycles suggests that the intervention may help modulate central sensitization or “pain memory” pathways (9). Ultimately, by providing profound analgesia during a critical painful period, AQLB could potentially interrupt cycles of anticipatory anxiety and pain amplification, leading to sustained symptomatic improvement (10). However, further studies are required to more fully elucidate these mechanisms.

This study has several limitations inherent to its case series design. First, the absence of a control group and the unblinded nature of the observations preclude definitive conclusions regarding treatment efficacy, as placebo effects cannot be ruled out. Second, due to variability in menstrual cycle length, the timing of follow-up assessments relative to the block differed across patients, which may have confounded the evaluation of analgesic duration. Third, although a standardized L4 approach was used, minor anatomical variations and differences in the timing of intervention relative to menstrual phase may have contributed to inter-individual variability in responses. These preliminary findings require confirmation through randomized controlled trials with sham comparators and standardized timing of intervention.

## Data Availability

The original contributions presented in the study are included in the article/supplementary material, further inquiries can be directed to the corresponding author.
